# Extended evolutionary psychology: the importance of transgenerational developmental plasticity

**DOI:** 10.3389/fpsyg.2014.00908

**Published:** 2014-08-20

**Authors:** Karola Stotz

**Affiliations:** Department of Philosophy, Macquarie UniversitySydney, NSW, Australia

**Keywords:** extended inheritance, parental effects, developmental niche, developmental plasticity, embodied cognition, extended cognition, extended evolutionary synthesis

## Abstract

What kind mechanisms one deems central for the evolutionary process deeply influences one's understanding of the nature of organisms, including cognition. Reversely, adopting a certain approach to the nature of life and cognition and the relationship between them or between the organism and its environment should affect one's view of evolutionary theory. This paper explores this reciprocal relationship in more detail. In particular it argues that the view of living and cognitive systems, especially humans, as deeply integrated beings embedded in and transformed by their genetic, epigenetic (molecular and cellular), behavioral, ecological, socio-cultural and cognitive-symbolic legacies calls for an extended evolutionary synthesis that goes beyond either a theory of genes juxtaposed against a theory of cultural evolution and or even more sophisticated theories of gene-culture coevolution and niche construction. Environments, particularly in the form of developmental environments, do not just select for variation, they also create new variation by influencing development through the reliable transmission of non-genetic but heritable information. This paper stresses particularly views of embodied, embedded, enacted and extended cognition, and their relationship to those aspects of extended inheritance that lie between genetic and cultural inheritance, the still gray area of epigenetic and behavioral inheritance systems that play a role in parental effect. These are the processes that can be regarded as transgenerational developmental plasticity and that I think can most fruitfully contribute to, and be investigated by, developmental psychology.

## Introduction

There exist two quite different stances toward the evolution of human cognitive capacities. The nativist stance, favored for instance by Evolutionary Psychologists (EP), attributes the origin of behavioral, social and cognitive capacities such as folk psychology, mind-reading and general reasoning capacities to the sudden appearance of genetically determined mental modules or representational systems. This approach, which subscribes to the computational theory of mind, has been polemically dubbed the “Rational Bubble stance (which) confounds cultural symbolic achievements with individual cognitive competences” and belongs to a class of views that have in recent years come under increasing criticism as a quite unrealistic model of cognitive growth (McGonigle and Chalmers, 2008, p. 143). An alternative view, now sometimes called the *e*mbodied, *e*mbedded, *e*nactive, extended (4E) cognition approach, is united by its opposition to traditional cognitivism and methodological individualism (Menary, 2010). Despite the differences between the separate views they all seem to agree on the necessity to place active agency at the center of cognition and the importance of cognition's scaffolding through developmental, ecological, and cultural niche construction. In addition this approach presupposes only very simple and modest biological preadaptations, e.g., in the perceptual realm or general developmental plasticity (Donald, 2000a,b; Griffiths and Stotz, 2000; Tomasello, 2000; Sterelny, 2003; Wheeler and Clark, 2008; Stotz, 2010).

There is natural affinity between one's view of the nature of the mind and an understanding of how the mind developed and evolved. The Modern Synthesis—the evolutionary theory to which EP is entirely wed—almost exclusively invokes natural (including kin and sexual) selection as the driving force, genetic mutations as the creative force and genetic transmission as the only mechanism of heredity. When applied to cognition, the Modern Synthesis invites the decomposition of the mind into separately evolved cognitive traits, called mental modules, each selected to solve a particular evolutionary problem in the human ancestor's “environment of evolutionary adaptedness” (Cosmides and Tooby, 1997). The origin of these modules is explained with the appearance of certain genetic factors that code for them. Strictly speaking the modern synthesis can be understood as a theory of genes, which arguably is poorly equipped to provide a more fully-fledged explanation of the transformation of form, other than the occurrence of genetic mutations and recombinations, which somehow translate into phenotypic modifications.

There is, however, a growing consensus that we need explanatory resources that go beyond inner logical representations or the dynamics of neural networks on the on hand, and the received view of evolution on the other. Physical, social and cultural life transforms individual characteristics and abilities in daily interactions, during individual development and the evolution of the lineage. The view of organisms, especially humans as deeply integrated beings embedded in and transformed by their genetic, epigenetic, behavioral, ecological, socio-cultural and cognitive-symbolic *legacies* calls for an “extended evolutionary synthesis” (Pigliucci, 2007, 2009; Pigliucci and Müller, 2010) that goes beyond either a theory of genes juxtaposed against a theory of cultural evolution or more sophisticated theories of gene-culture coevolution and niche construction. Environments, particularly in the form of developmental environments, do not just *select for* variation, they also *create* new variation by influencing development through the reliable transmission of non-genetic but heritable information (Piaget, 1978; Gilbert and Epel, 2009; Stotz, 2010; Griffiths and Stotz, 2013). Organisms, and humans in particular, are actively engaged with and manipulate their physical and social environment and that of their descendants, which in turn not only participates in the production of selection pressures, but almost more importantly in the production of heritable phenotypic variation; therefore organisms actively contribute to their own evolution. There is a strong interconnection between be highly embodied, embedded and extended cognitive systems and the kind of developmental and evolutionary processes that bring them about (Sterelny, 2010; Stotz, 2010).

The next section will explore the alternative views of evolutionary theory that provide much richer explanatory resources for (evolutionary) psychology. It will probe which updates to the conceptual structure of evolutionary theory would be needed for its most fruitful application to problems in psychology. The next two sections will explore in more detail two issues that are of particular importance for psychology. Section Beyond Innate and Learned: the Concept of Experience criticizes the conceptual poverty created by the simplistic dichotomy between innate or genetically determined development and acquired learning. It introduces another concept, experience, as a step to bridge between and integrate development and learning. Section Extended Inheritance discusses in more detail the diverse mechanisms of non-genetic inheritance that comprise the central extension to the Modern Synthesis.

The alternative conceptions of mind and cognitions to the traditional cognitivist and computationist approach, namely 4E cognition, will stand at the center of Section Embodied, Embedded, Enacted and Extended Organisms, Extended Synthesis and Extended Evolutionary Psychology. In this final section I look in more detail at how this alternative view of the mind is related to the extended view of evolution that I have promoted here, particularly those parts of the new synthesis that I deem to have special explanatory potential for many areas of psychology. A radically different perspective to the view that sees a disembodied mind being passively molded by natural selection and genetic mutations is presented. It conceives living beings as non-linearly coupled organism-environment systems, that come with cellular, social, ecological and cultural legacies bequeathed to them from earlier generations, and who's actions substantially influence the evolutionary process.

## Toward an extended evolutionary synthesis

### Kinds of evolutionary psychology

This section doesn't attempt to review, discuss and criticize in detail the main kinds of evolutionary psychology. There is an extraordinary amount of literature out there doing just that. This presents just a minimalist overview, and as such necessarily presents a bit of a caricature, the three waves of attempts to integrate psychology with evolutionary theory in the last four decades. In the seventies Edward O. Wilson attempted a “New Synthesis” under the name of Sociobiology, which viewed social behavior as the product of evolution and therefore reasoned that it should be explained in terms of adaptive success. Its arguably quite radical gene centrism, promoted particularly through Richard Dawkins' notion of the selfish gene, became the subject of intense criticism. Not entirely without intellectual connections, the nineties saw the emergence of the Santa Barbara school of Evolutionary Psychology. It applies knowledge and principles from mainstream evolutionary theory to psychology and “good old-fashioned” Artificial Intelligence/cognitive science in order to understand the design of the human mind: “In this view, the mind is a set of information-processing machines that were designed by natural selection to solve adaptive problems faced by our hunter-gatherer ancestors” (Cosmides and Tooby, 1997). This highly successful new field attracted a large number of followers over the years, but at the same time has been subjected to very vocal criticisms. Against EP's dismissal of the critics as politically motivated anti-evolutionists, many critics were biologists, philosophers of biology and psychologists motivated by a different vision of a more scientifically rigorous and a more sophisticated evolutionary psychology (see for instance Barrett et al., 2014).

The beginning of the century saw the appearance of evolutionary developmental psychology, applying evolutionary thinking to human developmental psychology: “Evolutionary developmental psychology is the study of the genetic and ecological mechanisms that govern the development of social and cognitive competencies common to all human beings and the epigenetic (gene-environment interactions) processes that adapt these competencies to local conditions” (Geary and Bjorklund, 2000, p. 57). This new field takes its inspiration from evolutionary developmental biology and so-called epigenetic theories of evolution going back to Gilbert Gottlieb in accepting a role for development, particularly developmental plasticity, in evolution (Bjorklund, 2006).

Which kind of evolutionary theory you apply matters deeply to which kind of (evolutionary) psychology you get. Therefore what follows will be a very short analysis of which kind of amendments or extensions should be included to widen the scope of problems that can be successfully addressed by evolutionary theory. Again, I have to refer to the cited literature for a much more thorough and detailed criticism and further amendment than I could present here; the present paper focuses on those new developments that in my view are most relevant for an extended evolutionary psychology.

This section asks what are the implications of recent scientific developments for the mechanisms of evolution. These developments include discoveries in molecular genetics, notably “molecular epigenesist” or “distributed specificity” of gene products, new discoveries of exogenetic heredity, and the revival of notions of epigenesis and developmental plasticity and their implications for evolution. Together with many others I have argued that these developments necessitate an extension of the conventional, neo-Darwinian theory of evolution, the so-called “Modern Synthesis.” Some may argue that the referral to the Modern Synthesis presents something like a straw man argument because many practitioners have, in the intervening decades, assimilated new conceptual and methodological developments into their thinking, often perhaps without being aware to what extent some of these violate the underlying assumptions on which the original synthesis was based. Several of these assumptions are now more than three quarters of a century old, and many of the relevant theories and concepts have undergone major revisions. So the point of the call for an “extended synthesis” may be as much to think through the implications of changes that have occurred or are occurring as to call for more change (Pigliucci, 2007, 2009; Craig, 2010; Pigliucci and Müller, 2010; Stotz, 2010; Gissis and Jablonka, 2011; Griffiths and Stotz, 2013).

### Molecular epigenesis

As stated above, the Modern Synthesis was in its core a theory of genes, but the gene that figures in it was the classical gene of Gregor Mendel and Thomas Hunt Morgan, a theoretical entity of heritable factors that permitted practitioners the “genetic analysis” of observed inheritance patterns. The knowledge of how genes conferred specificity within an organism and hence had observable phenotypic effects wasn't yet a molecular reality. Historians of molecular biology credit Francis Crick with having supplemented the existing idea of *stereochemical specificity*, embodied in the three-dimensional structure of biomolecules and underlying the well-known lock-and-key model of interaction between biomolecules, with the idea of *informational specificity*, embodied in the linear structure of nucleic acids (such as genes and other genetic elements) that determine the linear structure of a gene product (Sarkar, 1996). This idea is present in Crick's statements of his Sequence Hypothesis and the Central Dogma:

- *The Sequence Hypothesis* … In its simplest form it assumes that the specificity of a piece of nucleic acid is expressed solely by the sequence of its bases, and that this sequence is a (simple) code for the amino acid sequence of a particular protein.
- *The Central Dogma* This states that once “information” has passed into a protein it cannot get out again. In more detail, the transfer of information from nucleic acid to protein may be possible, but transfer from protein to protein, or from protein to nucleic acid is impossible. Information means here the precise determination of sequence, either of bases in the nucleic acid or of amino-acid residues in the protein. (Crick, 1958, pp. 152–153, italics in original).

Griffiths and Stotz (2013) have termed this encoding of specificity “Crick information.” If a cause makes a specific difference to the order of elements in a biomolecule, it contains Crick information for that molecule. This definition embodies the essential idea of Crick's sequence hypothesis, without in principle limiting the location of biological information to nucleic acid sequences as Crick does. An important idea behind Crick information is that this causally grounded notion of biological information can be extended to apply to factors other than DNA, using research results from molecular biology. Crick's Sequence Hypothesis and Central Dogma were based on a very simple picture of how the specificity of bio-molecules is encoded in living cells. We now know that, at least in eukaryotes, coding regions are surrounded by a large number of non-coding sequences that regulate gene expression. The discrepancy between the number of coding sequences and the sometimes vastly higher number of gene products leads to the insight that the informational specificity in coding regions of DNA must be amplified by other bio-molecules in order to specify the whole range of products. Different mechanisms of gene regulation together co-specify the final product of the gene in question, first by *activating* the gene so it can get transcribed, second by *selecting* a chosen subset of the entire coding sequence (alternative splicing), and thirdly by *creating* new sequence information through the insertion, deletion or exchange of single nucleotide letters of the RNA message (RNA editing). Thus specificity, and hence Crick information, is distributed between a myriad of factors other than the original coding sequence: Non-coding DNA sequences with regulatory functions, diverse gene products such as transcription, splicing and editing factors (usually proteins), and non-coding RNAs (Stotz, 2006a,b). This leads to the second substantive use of information in contemporary molecular biology, namely in representations of genomic regulatory networks (GRNs) as implementing computations (Kauffman, 1969; Davidson and Levine, 2005).

Specificity turns out to be not inherent in any single biomolecule in these large networks but induced by regulated recruitment and combinatorial control (Ptashne and Gann, 2002). And it is here that we will find that the networks cannot be reduced to DNA sequences and gene products, because many of the latter need to be recruited, activated or transported to render them functional. These processes, the recruitment, activation, location or transportation of transcription, splicing and editing factors, allow the environment to have very *specific* effects on gene expression. I believe that this is a way to give a more precise meaning to the distinction between “instructive vs. permissive environmental causes” (Gilbert, 2003; Gilbert and Epel, 2009). Many regulatory gene products serve to relay environmental (Crick) information to the genome. While in embryology and morphogenesis it is often acknowledged that environmental signals play a role in the organization of global activities; they are rarely seen to carry information for the precise determination of the nucleic acid or amino acid chains in gene products. But this is precisely what occurs. Not just morphogenesis at higher levels of organization, but even the determination of the primary sequence of gene products is a creative process of “molecular epigenesist” that cannot be reduced to the information encoded in the genome alone (Stotz, 2006b; Griffiths and Stotz, 2013).

Section Beyond Innate and Learned: the Concept of Experience will argue that these developments warrant the introduction of another concept in psychology, namely “experience,” beside the concepts of development and learning, to accommodate the many different avenues by which an organism reacts to and interacts with the environment.

### Non-genetic inheritance

Evolution is defined as changes in gene *frequencies*, because only the base sequence was deemed hereditary. The idea that developmental outcomes could be transmitted to the next generation was for almost a century discredited as raising the specter of Lamarckism. The molecular discoveries of epigenetic mechanisms, which produce effects on gene expression that were not just heritable from one cell to the other but in certain cases also from parent germline to offspring germline, however, have rendered ideas of non-genetic or exogenetic inheritance respectable. Parents beget their children not just gene *sequences* but also instructions over gene *expression* and other functional states. In addition, many aspects of the environment and individual experiences of a developing organism are there by evolutionary design: “genes *inherit* a rich and supportive environment, a fact few dispute but few discuss with any urgency” (West and King, 1987, p. 552, italics added). Evolution has designed not only a reactive genome, but also a “developmental niche” co-constructed by the parental and offspring generations to which the genome reacts to reconstruct life-cycles (see Section Developmental Niche Construction).

Since the development of molecular epigenetics has brought the existence of exogenetic inheritance to the attention of a much larger field, the idea is slowly gaining wider acceptance. The main points of debate today concern the scope and potential mechanisms of transgenerational transfer of non-genetic information, and its importance for evolutionary dynamics. Epigenetic inheritance proper, transmitted through the germline, and behaviorally transmitted transgenerational epigenetic effects (see Section Extended Inheritance) differ in several important ways from genetic inheritance: epigenetic variations may be less stable, because these variations are in principle reversible, and many organisms have developed safeguards against their transgenerational transmission. These features are not necessarily a disadvantage: in comparison to genetic inheritance, epigenetic mechanisms are more sensitive to the environment, which might make them more directed, more predictable, and also more flexible. These are all features which potentially render them more adaptive in the short term than blind genetic variation, particularly in variable environmental conditions (Jablonka and Lamb, 1995; Holliday, 2006). Exogenetic inheritance systems often transfer information involved in ‘adaptive transgenerational plasticity’ (see Section Transgenerational Developmental Plasticity: Parental Effects):

… because the parental phenotype responds to some aspect of its environment that correlates with a feature that is of adaptive relevance to the offspring. This correlational information can be exploited by developmental processes because of the continuity between parental and offspring phenotypes (…). In genetic inheritance systems, on the other hand, correlational information requires a process of selection that builds up gene frequency differences between environments. (Uller, 2012).

### Developmental plasticity and evolution

There are now a variety of scientific fields interested in the extent to which development influences evolution, and the ideas about which mechanisms are evolutionarily relevant differ greatly: The most radical position asserts that environmentally induced and developmentally regulated variation in exogenetic, developmental resources may be transmitted directly to the next generation either from germ or soma cell to germ cell, or from soma to soma, in order to create heritable variation in the phenotype (Griffiths and Gray, 1994; Jablonka and Lamb, 1995, 2005; Stotz, 2008, 2010; Stotz and Allen, 2008; Badyaev and Uller, 2009; Gilbert and Epel, 2009; Danchin et al., 2011; Bonduriansky, 2012; Uller, 2012).

Some biologists insist that only epigenetic inheritance transmitted through the gametes should be called a proper inheritance system, while the transmission from soma to germline or soma to soma is described as “transgenerational epigenetic effects” (Youngson and Whitelaw, 2008). Bonduriansky (2012) has argued that this resistance stems from the forceful association between genes and biological inheritance created by transmission genetics. For others less impressed by those historical developments transgenerational epigenetic effects are parental effects mediated via epigenetic mechanisms, which fall under the behavioral inheritance system (e.g., Meaney, 2001; Danchin et al., 2011; Griffiths and Stotz, 2013). Some evolutionary developmental biologists, traditionally reluctant to accept the existence of non-genetic inheritance, now embrace the importance of epigenetics for evolution, but see it as an extension of the genetic inheritance system since it works via the modification of the chromatin system, which forms part of the chromosome (Hallgrimsson and Hall, 2011).

According to others, phenotypically plastic responses during the lifetime (phenotypic accommodation) may uncover the existence, or facilitate the production, of suitable genetic change. Such genetic variation may lead via natural selection to either genetic assimilation (also known as the Baldwin effect), or genetic accommodation, in other words the genetic inheritance of either decreased or increased responsiveness to environmental conditions (Waddington, 1953a,b; West-Eberhard, 2003). Others argue that a group of conserved core processes of organisms facilitate the generation of phenotypic variation out of underlying genetic variation, called “facilitated variation” (Kirschner and Gerhart, 2010). Approaches closely related to the idea of facilitated variation maintain that evolution should be understood as a succession of developmental life cycles rather than change in gene frequencies, and it is therefore developmental mechanisms that provide the necessary causal explanations for how genetic change translates into phenotypic modifications (Hall, 1999).

Lastly, there are those within the niche construction (Odling-Smee et al., 2003) and the gene-culture coevolution approaches (Boyd and Richerson, 1985) who maintain that individual behavior and hence development influences evolution mainly by affecting the future selection pressure of the population through ecological and cultural niche construction activities. These modified selection pressures then feed back to evolutionary processes.

### Summary

The most important difference between the received view and an extended synthesis as conceived here is the acceptance of inheritance as a much wider phenomenon than originally understood. Inheritance is hence defined as the parental transfer to the next generation of all the developmental resources, including but not limited to DNA, that permit the reconstruction and modification of the developmental system. This developmental system is the whole organism-developmental niche complex. This reconstruction and modification encompasses both developmentally entrenched effects as well as sources for the expression of novel phenotypic variation, a case of potentially adaptive transgenerational plasticity. Some parental effects (see below) enable the persistence and even the spread of the induced phenotype by modifying selection pressure (e.g., when parental resources such as protection contribute to the offspring's ability to survive and reproduce).

So, while many still argue to what extent the transference of non-genetic resources influences population dynamics and the rate and direction of phenotypic evolution, here I want to advocate to want to advocate the extent to which it can provide more adequate answers to a wide range of central evolutionary questions (questions modified from Pigliucci and Kaplan, 2006). These include the:

- Origin of novel traits: adaptive transgenerational plasticity.
- Modification of traits: environmentally induced variability via parental effects.
- Spread of traits: the co-construction of a selective environment by developmental systems (selective niche construction).
- Maintenance of traits: stabilization of the developmental and selective niche.
- Reliable (re)production of traits: entrenched extended inheritance mechanisms (developmental niche construction) (compare Stotz, 2010).

## Beyond innate and learned: the concept of experience

Section Extended Inheritance will look in more detail at a range of mechanisms of, and fields of research into, non-genetic inheritance, in particular epigenetic inheritance both in its narrow and wider meaning, parental effects on offspring phenotype, and the idea of the developmental niche, which is constructed by these processes.

But before that, the root of some conceptual shortcomings in psychology—and beyond—needs to be addressed. This is the conceptual poverty expressed in the commonsense distinction between the innate and the acquired, usually decoded as caused by genes vs. being the product of learning (see Stotz and Allen, 2012 for a more detailed analysis of this problem). Unlike in biology, in wide areas of psychology the process of learning, instead of being understood as part and parcel of behavioral development, is set against the maturational, preprogrammed unfolding of the young to the adult.

A clarification of the relationship between the concepts of learning and development in psychology will require a *biologically informed* psychology, and the formulation of a broadened concept of “experience” may help to bridge the gap between learning and development by including all aspects of environmental stimuli that lead to long-term adaptive changes of behavior, including “learning” in its usual narrower sense. In other words, the concept of experience is not limited to sensory processing but includes a quite heterogeneous mix of environmental resources influencing the system's behavior. While this concept is not new, it unfortunately is not commonly used in scientific investigations, other than in its fields of origin (early comparative psychology and developmental psychobiology). My understanding of experience follows its original definition by the American animal psychologist Theodore Christian Schneirla, quoted by his student Daniel Lehrman: “Experience is ‘the contribution to development of the effects of stimulation from all available sources (external and internal), including their functional trace effects surviving from earlier development’ (Schneirla, 1957). Within this wide range of processes learning is only a relatively small part” (Lehrman, 1970, p. 30). To take this really on board one needs to acknowledge that physiological regulation and the regulation of behavior cannot be sharply separated, since their underlying mechanisms do not necessarily belong to distinctly different classes. This is especially so in early development. Reintroducing the concept of experience is not another way of saying that all behavior is learned, but a vehicle to bring home the inadequacy of the distinction between innate and acquired. It implicitly questions why “instinct” and “learning” should be the only two choices available to us for understanding behavioral development. A necessary requisite for the integration of the concepts of learning and development is to understand development as proposed by the developmental systems theory (Oyama et al., 2001).

The last decade has witnessed enormous scientific advances in genomics, systems biology, social neuroscience, evolutionary, and ecological and developmental biology (“evo-devo,” “eco-devo,” phenotypic plasticity, niche construction, extra-genetic inheritance, developmental systems theory). They challenge overly gene-centered and pre-deterministic as well as environmentalist explanations of behavior. Nature and nurture don't interact as if they were separated entities, with nature as the *a priori* plan being separated from concrete living and nurture being the means for modifying nature's plan through experience. Instead, every trait develops out of the nonlinear interaction between a range of very diverse developmental resources that cannot be usefully divided into genetic and non-genetic resources. It starts with the environmental regulation of gene expression, goes over a range of experiences beneath the skin and above the gene, over stages of sensory and social learning in vertebrates, to the exquisitely sensitive learning capacities of the human brain. “Nurture” is this ongoing process of development, while “nature” is the natural outcome of the organism-environment-system (Oyama, 1999).

Do we find learning or cognition in bacteria? The answer depends very much on your definition of learning and experience. Possibly yes, if “environment” is understood as the source of a “quite heterogeneous mix of resources called experience” (Moore, 2003, p. 350) extracted by a wide variety of means, only one of which is sensory, and if means for behavior derive from more than what is known to the senses. The concept of bacterial learning is no mere philosophical abstraction because of the many shared molecular pathways, often down to prokaryotes. For example, the NMDA receptors involved in the synaptic plasticity of neurons use proteins for binding amino acids that are highly conserved from bacteria (Kuryatov et al., 1994).

The study of behavior and cognition looks at three interconnected time-scales: evolution, development, and situated behavior. The integration of the first two seems possible now, and there are successful attempts at integrating the second two in areas of psychology, namely developmental psychobiology and social neuroscience (Michel and Moore, 1995; Cacioppo et al., 2002). This integration is based on an essential role for biology in psychology. From a psychobiological perspective, learning appears as a category within an overall framework of development as the lifelong, adaptive construction of the phenotype in its environment. Taking the idea of phenotypic plasticity seriously could, on the other hand, lead to a conception of development as a lifelong process of “learning” or “acquiring” an adaptive mode of living in a partially constructed environment.

## Extended inheritance

### Mechanisms of transmissions: four inheritance systems

Eva Jablonka and Marion Lamb propelled the idea of epigenetic inheritance to prominence with their provocative title *Epigenetic Inheritance and Evolution: The Lamarckian Dimension* (Jablonka and Lamb, 1995). Epigenetic inheritance in this context is used primarily in its narrow sense as the inheritance of cellular functional states via structural elements (e.g., membranes), steady-state systems (self-perpetuating metabolic patterns), and chromatin modification (chemical modifications of histone proteins or DNA bases), although it often spills over into a broader sense to include other exogenetic inheritance systems. Jablonka and Lamb's identification of epigenetic inheritance with the inheritance of acquired characters is not unproblematic. Some scientists insist that the term Lamarckian inheritance should be restricted to the inheritance of phenotypic (somatic) characters that are acquired during development (Hall, 2011, p. 11). It would also entail a directed response to the environment, not just blindly caused by it. While strict epigenetic inheritance is transmitted through the germline, it is often mixed up with “experience-dependent epigenetic inheritance” (Danchin et al., 2011) in the broader sense, which should really be understood as behavioral and ecological inheritance mediated by epigenetic effects. As said above, the latter form has also been termed transgenerational epigenetic effects (Youngson and Whitelaw, 2008). The problem may often be that the exact underlying mechanism for a parental effect is not yet known. Epigenetic inheritance of the latter kind may also have distinctive evolutionary advantages. Particularly the parental effect literature offers a wide range of examples where parental effects, that work later in development and are mediated by the latter kind of epigenetic effects, enable the development of functional phenotypes in the offspring (Uller, 2012).

Some molecular biologists have argued that one should speak of epigenetic inheritance in the literal sense only in those cases when the methylation pattern is transmitted unchanged over several generations (Wilkins, 2011, p. 391). Some cases certainly meet this criterion. In a comprehensive review of epigenetic inheritance Jablonka and Raz conclude that it is ubiquitous, and can show stability of transmission of up to 3 generations in humans and up to 8 generations in other animal taxa, while plants can have a very stable epigenetic transmission (Jablonka and Raz, 2009). Many cases, however, would indeed not meet the criterion of multi-generational transmission. Epigenetic signals are very sensitive to environmental factors in that they are first “established by transiently expressed or transiently activated factors that respond to environmental stimuli, developmental cues, or internal events” (Bonasio et al., 2010, p. 613). That doesn't mean that we should accept the criterion of multi-generational stability. Several hypotheses about the evolutionary origins of epigenetic inheritance stress its value in spatially and temporally heterogenous environments, where it allows rapid responses to change. It is simply not correct that epigenetic change will only affect evolution if the changes themselves persist for more than one generation. Parental effects researchers have long known that one-generation parental effects substantially alter the dynamics of evolutionary models by changing which equilibrium a population will evolve to (Wade, 1998). In conventional quantitative genetics, the importance of Mendelism is not that individual genes can be tracked from one generation to the next—quantitative genetics does not do this—but that Mendelian assumptions let us work out what phenotypes (and hence their fitness) will appear in the next generation as a function of the phenotypes in the last generation. Epigenetic inheritance changes that mapping from parent to offspring, and this will affect evolution. There is no more central instance of the study of heredity than quantitative genetics, so more argument is needed for why epigenetic inheritance needs to be stable for several generations to be regarded as a form of heredity.

As I mentioned above, discussion of epigenetic inheritance often spills over from discussion of the specific phenomena of meiotic inheritance of chromatin modifications to include other phenomena that produce a parental effect. This is understandable, because molecular epigenetic mechanisms are often important in parental effects that do not involve actual epigenetic inheritance. For example, in one well-studied example, epigenetic mechanisms have been shown to mediate the transgenerational effect of maternal care in rats without actual epigenetic inheritance. Maternal behavior establishes stable patterns of methylation in the pups. These affect brain development and the behavior of the next generation of mother rats. While the behavior of those mothers reestablishes the patterns of methylation, they are not inherited through the germline (Meaney, 2001; Champagne and Curley, 2009). So long as the environment is constant, or the epigenetic pattern is maintained throughout the lifetime of the parent and reliably programs parental behavior, the phenotype will remain constant through many generations. The authors call this environmental programming of certain types of behavior through DNA methylation “life at the interface between a dynamic environment and a fixed genome” (Meaney and Szyf, 2005).

In a recent book, Jablonka and Lamb have attempted to organize the topic of epigenetic inheritance in this wider sense around four “dimensions” of heredity: Genetic, Epigenetic, Ecological, Behavioral and Cultural, and Symbolic (Jablonka and Lamb, 2005). The Genetic Inheritance System comprises protein coding and non-coding RNA genes plus the regulatory motifs in the genome, as well as sequences with unknown functions. The Epigenetic Inheritance System includes modifications of DNA and chromatin, which are part of the nucleus. Beside these resources that are literally physically attached to the genome other developmental resources are transmitted through the cytoplasm of the egg, such as parental gene products (regulatory proteins and non-coding RNAs). The cortical inheritance system consists of cellular structures such as organelles with their own membranes and genes (mitochondria and chloroplasts), membrane-free organelles (ribosomes and the Golgi apparatus), and the cellular membrane systems. Most of these structures cannot be produced from genetic information alone but act as templates for themselves. A Behavioral (plus cultural and ecological) Inheritance System forms a third dimension, in which information is transmitted through behavior-influencing substances, non-imitative and imitative social learning, as well as habitat construction, food provisioning, and other parental effects like that described in the last paragraph. The Symbolic (plus the Cognitive) Inheritance System forms the last dimension. Offspring inherit social structures and rules, cultural traditions and institutions, and technologies. This inheritance system importantly includes epistemic tools, such as language, competent adults, teaching techniques etc. (compare Jablonka and Lamb, 2005). All systems use different mechanisms of transmission and show changing degrees of fidelity. Some mechanisms may not be intrinsically stable. The nuclear genetic inheritance system, for example, relies on several layers of proof reading and copy-error detection systems for its exceptionally high fidelity. A suitable mechanism of scaffolding can lend the transmission mechanism reliability: proof reading supports genetic inheritance, epigenetics stabilizes gene expression. Learning is scaffolded by teaching or by the reliable affordances of stimuli “that define what is available to be learned … (and) … function to channel malleability into stable trajectories” (West et al., 2003, p. 618).

As already mentioned, apart from its quite clearly defined molecular sense, epigenetic inheritance can also mean something much more general and much less clearly delineated. This other meaning derives in part from Waddington's original understanding of epigenetics, but also tries to integrate newer developments and understanding:

Epigenetics … focuses on the general organizational principles of developmental systems, on the phenotypic accommodation processes underlying plasticity and canalization, on differentiation and cellular heredity, on learning and memory mechanisms. Epigenetics includes the study of the transmission to subsequent generations of developmentally-derived differences between individuals, thereby acknowledging the developmental aspect of heredity. (Jablonka, pers. comm., cited in Gottlieb, 2001).

### Transgenerational developmental plasticity: parental effects

Amongst the oldest of the research agendas investigating processes of transgenerational transmission of nongenetic resources is work on “parental effects.” As its name suggests work of this sort does not start from findings about underlying mechanisms. Instead, it begins with the relationship between parent and offspring phenotypes. Parental effects are sustained influences on offspring phenotype that are derived from the parental phenotype beyond the nuclear genes bequeathed to the offspring. The parental phenotype is the result of genetic, environmental and (grand-) parental effects, and their interaction. More formally, we can say that a parental effect is a correlation between offspring and parent phenotypes that is statistically independent of the correlation between their genotypes.

Parental effects are received as part of the environmental component of offspring phenotypes. The environment provided by the mother for her offspring is a very important factor in causing fitness differences among newborns and weanlings, particularly in organisms with extensive parental care. In environmentally induced parental effects the environment experienced by the parental generation influences the phenotype of the offspring. In locusts, an environment overcrowded with conspecifics experienced by the mother causes her to coat her eggs with a hormonal substance containing serotonin, which induces the egg to develop into a high-density morph with wings and legs suitable for migration. Many parental effects, like this one, enhance the offspring's fitness. Natural selection has shaped offspring to respond to subtle variations in parental behaviors or parental provisioning as a forecast of the environmental conditions they will ultimately face after independence from the parent (Mousseau and Fox, 1998; Maestripieri and Mateo, 2009). The organism's developmental plasticity utilizes environmental cues or developmental resources inherited from the parents to fine-tune its phenotype to the current or expected environment.

Because parental effects are defined phenomenologically—an observable relationship between phenotypes, any mechanism that produces this relationship counts as a parental effect. The domain of phenomena called parental effects includes narrow-sense epigenetic effects that are reproduced in meiosis and thus can pass from one generation to another, but it includes many other things as well. The mechanisms that can create a parental effect include: parental gene products (mRNAs, ncRNAs, proteins); cytoplasmic inheritance (mitochondria, plastids, membranes, signaling factors, chemical gradients, intra-cellular symbionts; often investigated separately as maternal inheritance); oviposition (the placement of eggs in insects, fish, and reptiles can effect food availability and quality, temperature and light conditions, and protection against predators and other adverse conditions, and hence has important consequences for the fitness of the offspring); gut organisms (which are often necessary for the normal development of intestines and the immune system, and daily metabolism); sex determination (via maternal influence on temperature exposure in reptiles, hormonal influence on gamete selection in birds); nutritional provisioning (prenatally through seeds, eggs, and placenta, postnatal feeding particularly in mammals and birds, that not only provides sustenance for the offspring but influences later food preferences, feeding behavior, and metabolism); parental care and rearing practices (warmth, protection, and emotional attachment, e.g., differential licking in rats, teaching and learning); social status (in hierarchically organized mammals, such as primates, offspring often inherit the social status of the mother), among other things (Mousseau and Fox, 1998; Maestripieri and Mateo, 2009). Although most of these phenomena do not count as narrow-sense epigenetic inheritance, because they do not involve the transfer of chromatin modifications through meiosis, the phrase “epigenetic inheritance” is sometimes used in a wide sense that is more or less equivalent to parental effects. The reason is that they often assert their effect on the phenotype via epigenetic mechanisms caused by the maternal phenotype. I prefer to use the less ambiguous phrase exogenetic inheritance in those contexts where the exact underlying mechanisms are not yet known.

As might be expected from such a diverse field, there are many different approaches to parental effects. Parental effects researchers Badyaev and Uller (2009) have shown how the differences in the ways parental effects are understood reflect the different roles they play in research. These different approaches do not necessarily count exactly the same phenomena as parental effects. For many geneticists it is essentially a statistical concept, i.e., an additional parent-offspring correlation that must be added to a quantitative genetic model in order to correctly predict the effects of selection. In contrast, someone studying animal development is likely to define parental effects at a mechanistic level, referring to specific ways in which they are produced. Evolutionary biologists see parental effects either as adaptations for phenotypic plasticity, or as the consequence of a conflict between parent and offspring seeking to influence each other's phenotype to suit their own interests:

… parental effects mean different things to different biologists—from developmental induction of novel phenotypic variation to an evolved adaptation, and from epigenetic transference of essential developmental resources to a stage of inheritance and ecological succession. (Badyaev and Uller, 2009, p. 1169).

I suggest that the distinctive feature of parental effects is that it is a phenomenological concept. So parental effects should not be defined by any specific mechanism that brings them about. Second, parental effects should not be defined as adaptations, since their evolutionary significance does not depend on this—the correlations have the same impact on the dynamics of evolution whether or not they are adaptations. From a developmental perspective, parental effects need to be understood before the difficult question of their evolutionary origins can be properly addressed. More importantly, non-genetically inherited resources shouldn't be understood as competing with genetic resources; they complement them. They do this in part by amplifying the sequence information encoded by nucleic acids, as summarized by the idea of molecular epigenesis. Badyaev and Uller summarize the significance of parental effects in development and evolution very nicely:

Here, we suggest that by emphasizing the complexity of causes and influences in developmental systems and by making explicit the links between development, natural selection and inheritance, the study of parental effects enables deeper understanding of developmental dynamics of life cycles and provides a unique opportunity to explicitly integrate development and evolution. …parental effects on development enable evolution by natural selection by reliably transferring developmental resources needed to reconstruct, maintain and modify genetically inherited components of the phenotype. The view of parental effects as an essential and dynamic part of an evolutionary continuum unifies mechanisms behind the origination, modification and historical persistence of organismal form and function, and thus brings us closer to a more realistic understanding of life's complexity and diversity (Badyaev and Uller, 2009, p. 1169).

### Developmental niche construction

The concept of the ontogenetic niche was introduced in 1987 by developmental psychobiologists Meredith West and Andrew King. It provides a way to bring together the research agendas described above that focus on exogenetic inheritance mechanisms in the widest sense. Many aspects of the environment and experience of a developing organism are there by design: Evolution has designed not only a reactive genome, but also a developmental niche that reacts with it to construct phenotypes. West and King define the ontogenetic niche as a set of ecological and social circumstances inherited by organisms (West and King, 1987, p. 550). One should add epigenetic, epistemic, cultural, and symbolic legacies to this list and point to Jablonka and Lamb's “dimensions” of heredity as a thorough and principled effort to taxonomize the contents of the developmental niche (Jablonka and Lamb, 2005; Stotz, 2006c, 2008, 2010; Griffiths and Stotz, 2013). Naturally, some dimensions are more prominent in one taxon than another. Together, these legacies are designed to provide the developmental resources needed to reconstruct and modify the life-cycle in each generation. The developing organism can expect to encounter this niche in development as reliably as it does its genome: “It's the dependability of the niche in delivering certain resources to the young that makes it a legacy” (West et al., 1988, p. 46).

The developmental niche provides an alternative to the nature-nurture dichotomy (Stotz, 2008; West and King, 2008). The niche equals nurture since it nurtures the developing organism, and it equals nature (traditionally understood as the innate), because it is part of the organism's endowment. West and King and their collaborators devoted decades of painstaking research to the acquisition of species-typical behavior of the Brown-headed Cowbird. As a nest parasite the cowbird had been assumed, since it could not learn species-specific behaviors from its parents, to inherit those behaviors genetically: they are innate. West and King set out to show that this kind of dichotomous thinking was no substitute for a causal analysis of how the phenotypes actually develop. The results of this research led them to develop the “ontogenetic niche” concept. The ability of cowbirds to recognize their own species visually depends, amongst other factors, on “phenotype matching”—individuals seek to interact with birds that look like themselves. This, in combination to ecological factors, helps ensure that cowbirds find themselves in flocks. Male song is shaped by feedback from female cowbirds, whose wing stroking and gaping displays in response to the songs strongly reinforces males (West and King, 2008). Raised in isolation males will sing, but they need feedback from a mature female audience and also competition with other males in order to learn how to produce cowbird songs in a way that lead to successful mating:

In cowbirds the juvenile niche is a forum in which males learn the pragmatics of singing, which appears to be a performatory, if not sometimes martial, art. (West and King, 1987, p. 52).

Female song preferences are themselves socially transmitted. As a result, cowbirds reliably transmit not only species-typical songs, but also regional song dialects. The flock functions as an information center, controlling what is “bioavailable” to be learned throughout the lifespan. The developmental niche concept undermines the traditional dichotomy between heredity and individual experience, since it highlights how experience, including in some taxa real social learning, is involved in the development of species-typical behavior. Aspects of experience are part of the mechanism of heredity (West and King, 2008).

The cowbird is not an isolated example. Other examples in which developmental niches afford the robust experiences necessary for normal development include food and habitat imprinting in insects through oviposition; maternal care and stimulation for neural development (sexual behavior and fear reaction in rats; learning disposition in chickens); territorial and habitat inheritance (nest sites, food resources, a hierarchy of relatives) in woodpeckers and jays; maternal rank inheritance in carnivores and primates (Maestripieri and Mateo, 2009).

Jeff Alberts has used the developmental niche extensively in studies of rat development. The rat pup passes through four consecutive “nurturant niches” on the way to adulthood: the uterine niche, the dam's body, the huddle in the natal nest, and the coterie (Alberts and Schank, 2006). They all provide sustenance for the developing organism, such as nutrients, warmth, insulation, and “nurture” in the form of behavioral and social stimuli as affordances for development. The early ontogeny of species-typical rat behavior is directed mainly by olfactory, but also tactile, cues that are provided by the different ontogenetic niches. Olfactory cues on the dam's nipples guide the pup to them. However, the pup's developing sensoria need to acquire odor recognition of the nipple through chemical cues in the amniotic fluid provided by the uterine niche it had passed through before. The spread of amniotic fluid over the dam's body after birth bridges the pre- and postnatal niches of the pup. Filial huddling preferences in the natal niche are mediated by learned olfactory cues provided from the close proximity of the siblings during the suckling stage. This huddle or natal niche in turn induces preferences prerequisite for the functioning of the rat in the social context of the “coterie niche,” through thermotactile stimulation. Alberts notes:

Again we find a stereotyped, species-typical, developmentally-fixed behavior is learned, with all of the key components […] existing as natural features of the ontogenetic niche. … Specific features of these niches elicit specific reactions and responses in the developing offspring. (Alberts, 2008, p. 300).

These niches afford the pups a range of other experiences. In the previous section we encountered Michael Meaney and collaborators' discovery that natural variation in maternal care, elicited by experiences of the mother, influence stress responses, exploratory and maternal care behavior in the offspring. The quality of the mother's licking and grooming behavior results in a cascade of neuro-endocrine and epigenetic mechanisms. One pertinent example is the permanent down-regulation in the expression of the glucocorticoid receptor gene in the pup brain's hippocampus via the methylation of its promoters, which occurs in response to a low-level of licking and grooming by the mother (Meaney, 2001; Champagne and Curley, 2009). This down-regulation causes high stress-reactivity in the offspring. Hence stressful mothers in reaction to an adverse environment produce stressful daughters who in turn become stressful mothers. This is not necessarily bad, since highly stressed individuals are better prepared to survive in adverse environments (e.g., a high level of predation). Conversely, relaxed mothers that show a high level of licking and grooming produce relaxed offspring that turn into high licking mothers. Experiences can help to construct the legacy that the next generation will receive: “Exogenetic legacies are inherited, but they are also learned” (West et al., 1988, p. 50).

The developmental niche explains the reliable development of species-typical features, but the framework is equally applicable to plastic phenotypes. Many developmental systems are “designed to be as open as ecologically possible and thus immediately sensitive to ecological change” (West and King, 2008, p. 393). The niche contains the scaffolding for normal development, but the genome has coevolved with the niche and can also use it as a source of information for developmentally plastic responses: “Animals have evolved to integrate signals from the environment into their normal developmental trajectory” (Gilbert and Epel, 2009, p. 9). The fact that development is not laid out before it occurs, with other causal factors as merely permissive (or disruptive), but instead emerges through a process of epigenesis, is what enables the integration of robustness and plasticity in development (Lamm and Jablonka, 2008; Bateson and Gluckman, 2011).

## Embodied, embedded, enacted and extended organisms, extended synthesis and extended evolutionary psychology

In the introduction I alluded to the interplay between the nature of the organism and mechanisms of evolution. This section will explore this relationship in more detail. While what kind of mechanisms one deems central for the evolutionary process deeply influences one's understanding of the nature of organisms, adopting a certain approach to the nature of life and cognition and the relationship either between the two or between organism and environment should affect one's view of evolutionary theory.

One perspective views species of organisms as passively molded by external selection working on the random mutations organisms possess, with the evolutionary process pretty much unaffected by the behavior of organisms themselves (or so the equations at the heart of evolutionary theory, population genetics, imply). A radically different perspective views living beings as non-linearly coupled organism-environment systems, whose actions substantially influence the evolutionary process via two different pathways: the developmental creation of potentially directed or adaptive variations, and the active construction of the population's niche and hence the selection pressures acting on it. Under the latter perspective it shouldn't matter if you start with a revised vision of the nature of life and cognition or a revised vision of the nature of the mechanisms that underlie the process of evolution. One should prescribe the other.

### Disembodied minds and the modern synthesis

The modern synthesis asserts that mutation in genes provide the only (and, at that, blind and non-directed) variations and the slow process of natural selection acting on these variations is its direction-giving, order-producing or creative force. Genetic variations determine the organism's characteristics, which in turn influence its fitness, i.e., its ability to survive and reproduce. Genetic inheritance renders these fitness differences hereditary. Since evolution is defined as changes in gene frequencies, it is implied that development, including potential developmental modifications through phenotypic plasticity or the behavior of organisms, plays no role in this process. The causes of phenotypic variation, evolution's main currency, are solely genetic: blind mutation, sexual recombination, genetic drift and gene flow. Additionally, natural selection is the result of hostile external forces on populations, a “struggle for existence” which results in the “survival of the fittest.” Both take place without any acknowledged influence of the organism—with the notable exception of sexual selection, already envisioned by Darwin as a secondary mechanism of selection, which is greatly influenced by the choice of an organism's choice of sexual partners. In the seventies, ideas of evolutionary game theory, the competition between different genetically inherited strategies of survival and reproduction, were added. Today we also have theories of coevolution, such as gene-culture coevolution, and niche construction. These theories all work by recognizing that populations influence, to some extent, their own selection pressures. The main reason for this relative neglect of the organism in the modern synthesis was that any influence by the developing organism that is not originally caused by its genetic make-up was assumed to not leave any trace effects on its descendants. And so it comes as no surprise that the main equations in population genetics, the formal backbone of the modern synthesis, calculate frequency and interaction of alleles and genes in populations, without any mention of the organism.

### The role of embodied, embedded and extended organisms in an extended evolutionary psychology

The adapted mind of evolutionary psychology has a cognitive foundation of innate, content-rich mental modules, supposed to be universally shared by all humans. EP proposes that strong selective pressures during the ancestral environment of evolutionary adaptedness (EEA) have accounted for the evolution of these faculties, shaped by environmental problems of the human's ancestor in the Pleistocene. They include not only sets of rules and algorithms for problem-solving and other mental faculties, but consist of concrete information reflecting the structure of the real world at the time when our mental capacities were meant to have evolved.

The alternative view of evolution as the “change in the distributions and constitution of developmental systems” does not posit a population of static and passive entities. Instead of random mutations and drift, developmental plasticity produces variable and active organisms that engage with their environment. The core of heredity, one of the main supporting beams of the received view, doesn't have to be the persistence of traits due to un-interrupted channels of genetic transmission. Instead, self-organizational properties of the system actively create and construct both stability (heredity) and variability (adaptability) through the availability of developmental resources in every generation. Table 1 describes the main tenets of an Extended Evolutionary Synthesis as sketched earlier in the paper.

**Table 1:** **A comparison between the modern and the extended synthesis**.

**Evolutionary mechanism**	**Modern synthesis**	**Extended synthesis**
Variation	Mutation, recombination	Mutation, recombination, developmental plasticity, variability
Inheritance	Genetic inheritance	Genetic (incl. assimilation and accommodation), epigenetic, ecological, behavioral/cultural, symbolic inheritance; parental effects, developmental niche construction
Natural Selection/adaptation	Independent external force on population	Niche construction Complex adaptive systems; adaptability; evolvability
Adaptation	Genetic solution to environmental problem	Active mind in active body embedded in world
Development	Genetic program	Interactive construction, developmental niche
Organism	Passive object in hostile world	Active agent in own evolution

As argued here, an extended evolutionary theory is reciprocally related to the view of a cognitive system as embodied, embedded, enacted, and extended, promoted recently by many proponents in cognitive science. There are plenty of older thinkers who have promoted similar ideas and present therefore part of the heritage of this view^1^. For the particular argument in this section, and indeed this paper, which are related to the relationship between biological development and evolution with a view of cognition, a very important proponent has been Jean Piaget. His main subject of study was the origin of knowledge:

Of course the most fruitful, most obvious field of study would be reconstituting human history—the history of human thinking in prehistoric man. … Since this field of biogenesis is not available to us, we shall do as biologists do and turn to ontogenesis. (Piaget, 1970, p. 13).

In painstaking psychogenetic studies, Piaget established that organisms are not passive achievers of knowledge or reactors to external conditions; to the contrary, the system seeks its own experience and reacts to stimuli with active and creative changes in itself and in the environment. Piaget's genetic epistemology gave a plausible explanation for the relation between cognition and action (as the Santiago School of Maturana and Varela put it: no cognition without action, and no action without cognition). Piaget emphasized the emergence of cognitive abilities out of a groundwork of sensorimotor abilities. At the beginning of an interaction there is no *subject* and no *object*. Both result from the internal organization of the subject's experience with the external. For Piaget, boundaries between the cognitive subject and the outside object were not *given* qualities but *created* categories of the world. The cognitive system was the subject-environment unity. Piaget attempted to show the constructivist-interactionist power between the two antagonistic mechanisms of life: organization and adaptation - maintenance and change - essential for every natural process, like development, evolution, and cognition. Development, in this view, is the construction of all kinds of causal factors—genetic, epigenetic, ecological, social and cultural factors—which interact together in a self-organized and not centrally controlled manner (Stotz, 1996). His understanding of ontogenesis hugely influenced his conception of evolution. Piaget was one of the first to take a developmental perspective on evolutionary processes. His genetic epistemology was a biology of knowledge based on an evolutionary theory situated between the extremes of (neo-) Darwinism and Lamarckism. Behavior is seen as a driving force in evolution, and an adaptation to the environment is understood as the result of an interactive construction of self and the environment.

Before going through the main tenets of evolutionary theory, variation, heredity, selection and adaptation, and the role the active organism is playing in all of them, we first have to address the very preconditions for biological evolution. Organisms as they exist today may owe their existence to evolution, but the evolutionary process in turn crucially depends on the existence of living systems that exhibit the necessary preconditions, namely the capacity to (a) produce variation (variability), (b) transmit information for the reconstruction of the life cycle to the next generation (heritability), and (c) adapt the behavior to the contingencies of the environment (adaptability). In summary, populations of organisms must exhibit the ability to evolve, the production of heritable variation in fitness, i.e., differential reproductive success (evolvability). These preconditions, if fulfilled, produce natural selection. Organisms are not just actively engaged in the business of being alive—“acting on their own behalf” (Kauffman, 2000), they are at the same time creating the pre-conditions for evolution, and actively shape the evolutionary process.

There are two main ways they do so: the developing organism can modify the selection pressure acting on the population via the processes of niche construction (Odling-Smee et al., 2003) and cultural evolution (Boyd and Richerson, 1985); or the developmental system can actively create evolutionary novelties and variation, since new variations are the raw material of evolution. For Piaget the “central problem remains”: is the environment through its influence on development and behavior “also a causal factor in the actual formation of morphological characteristics” (Piaget, 1978, p. xi)? The extended evolutionary synthesis entails the belief that “the environment not only selects variation, it helps construct variation” (Gilbert and Epel, 2009, p. 369). Hence, the second way to shape the evolutionary process is by creating different and interacting channels of heredity with varying degree of reliability and adaptive plasticity that help to recreate and modify the life cycles of the next generations.

The hypothesis of molecular epigenesis, which could only be sketched here in a rough outline but is substantiated by 20 years of experimental research, very strongly supports the developmental plasticity exhibited by almost all organisms in a more or less extensive degree. Hence the developmental process, during which the organism is in a very tight relationship to its developmental environment, is potentially a very important contributor to heritable variation.

Recent theories in cognitive science have begun to focus on the active role of organisms in exploring and shaping their own environment, and the role of these environmental resources for cognition. Within cognitive science, with its long history of interpreting the mind as a disembodied symbol-processing machine, 4E cognition has been treated as quite a radical departure. Approaches such as situated, enacted, embodied, embedded, ecological, distributed and particularly extended cognition look beyond “what is inside your head” to the old Gibsonian question of “what your head is inside of” and with which it forms a wider whole—its internal and external cognitive niche. Similar embodied and extended views have been proposed within (philosophy of) biology, most notably Developmental Systems Theory and the theories of (selective) and developmental niche construction.

These two views are sometimes seen as mere analogies to each other. From the view of embodied and enacted cognition, in particular, this should immediately be seen as a mistake. The developmental construction intimately relates to the construction of cognition, since biological brains are the control systems for biological bodies. Cognition is the organism's permanent interaction with the world. Even more so in organisms with complicated brains while interacting with the world they permanently construe this world, which in turn influences its impact on development. The mind can only be understood in the context of its relationship to a physical body, which allows it to interact with the world. This world in turn is part of the biological and cognitive system. The body or the mind alone is often not a meaningful unit of analysis because of the dense and continuous information flow between mind, body and world.

The relationship between an active developing system with exploring cognitive abilities and its capacity to construct its own living environment—and that for its descendents—becomes immediately obvious. Since the organism-environment relationship creates selection pressures that will have an important feedback on the phenotype of future generations, the organism indirectly and partially controls its own evolution. This argument has been extensively developed and defended by Odling-Smee, Laland and Feldman (e.g., 2003). The important role of niche construction and scaffolded or extended cognition particularly for human evolution has also been widely argued for (e.g., Sterelny, 2003, 2010; Wheeler and Clark, 2008). But the relationship of the active organism, embedded in its environment, and developmental plasticity to create evolutionary variation and innovations crucially depends on its ability to make these modifications heritable.

Only quite recently did new mechanisms come to light that showed to what extent heredity is indeed a developmental achievement, as the embryologists of the nineteenth century have argued. First, epigenetic mechanisms inherited through the germline made the idea more palatable to biologists, for whom any transmission outside the germ line was previously principally excluded from consideration. There were earlier hypotheses, notably genetic assimilation and accommodation, that that afforded explanation seemingly Lamarckian phenomena by genetic inheritance, widely construed. But since inheritance has now been somewhat detached from its dogmatic relationship with the genetic system, it becomes possible to interpret other transgenerational transmissions as inherited. Parental effects have long been recognized as a main contributor to the offspring phenotype. This phenotype develops out of contributions received from the parents (both genetically and behaviorally) and environmental contributions. Why should some of these trans-generationally transmitted resources not be accepted as contributing to the inheritance of the offspring, as long as they influence the dynamics of evolution?

But all of these exogenetic contributions of inheritance rely critically on the active organism embedded in its own niche, namely caring for its offspring (like the nutritional contribution to the egg, the positioning of the seeds, and parental care in birds and mammals), and the organism's complex relationship with its offspring that actively co-construct the offspring's developmental niche. Epigenetic, behavioral, ecological, and epistemic inheritance depends on the environment experienced and provided by the parent.

This paper couldn't possibly focus on all aspects of an extended evolutionary synthesis and has chosen the, from the author's perspective, most important but also most highly debated aspect, namely non-genetic inheritance. Within this area the paper has barely touched cultural inheritance because most papers on extended inheritance and its importance particularly for human development and evolution has focused on that. Instead it stressed particularly those aspects of extended inheritance that lie between genetic and cultural inheritance, the still gray area of epigenetic and behavioral inheritance systems that play a role in parental effects. These are the processes that can be regarded as transgenerational developmental plasticity and that I think can most fruitfully contribute to, and be investigated by, an extended evolutionary psychology.

### Conflict of interest statement

The author declares that the research was conducted in the absence of any commercial or financial relationships that could be construed as a potential conflict of interest.

## Footnotes

^1^There are indeed many differences between the different views, such as between enacted and extended, or embodied and *radical* embodied cognition. For the argument of this paper, however, the many facets of these views and where and how they disagree with each other should not concern us.
